# Radiation endurance in Al_2_O_3_ nanoceramics

**DOI:** 10.1038/srep33478

**Published:** 2016-09-22

**Authors:** F. García Ferré, A. Mairov, L. Ceseracciu, Y. Serruys, P. Trocellier, C. Baumier, O. Kaïtasov, R. Brescia, D. Gastaldi, P. Vena, M. G. Beghi, L. Beck, K. Sridharan, F. Di Fonzo

**Affiliations:** 1 Center for Nano Science and Technology @PoliMi, Istituto Italiano di Tecnologia, Via Pascoli 70/3, 20133 Milano (MI), Italia; 2 Department of Engineering Physics, University of Wisconsin-Madison, 1500 Engineering Drive, 53715 Wisconsin (WI), USA; 3 Smart Materials, Nanophysics, Istituto Italiano di Tecnologia, Via Morego 30, 16163 Genova (GE), Italia; 4 Laboratoire JANNUS, DEN-Service de Recherches de Métallurgie Physique, CEA, Université Paris Saclay, F-91191 Gif-Sur-Yvette, France; 5 CNRS/IN2P3/CSNSM/SEMIRAMIS/JANNUS-Orsay, Université Paris Sud, Bat. 108, 91400 Orsay, France; 6 Department of Nanochemistry, Istituto Italiano di Tecnologia, Via Morego 30, 16163 Genova (GE), Italia; 7 Dipartimento di Chimica, Materiali ed Ingegneria dei Materiali, Politecnico di Milano, Via Mancinelli 7, 20131 Milano (MI), Italia; 8 Dipartimento di Energia, Politecnico di Milano, Via Ponzio 34/3, 20133 Milano (MI), Italia

## Abstract

The lack of suitable materials solutions stands as a major challenge for the development of advanced nuclear systems. Most issues are related to the simultaneous action of high temperatures, corrosive environments and radiation damage. Oxide nanoceramics are a promising class of materials which may benefit from the radiation tolerance of nanomaterials and the chemical compatibility of ceramics with many highly corrosive environments. Here, using thin films as a model system, we provide new insights into the radiation tolerance of oxide nanoceramics exposed to increasing damage levels at 600 °C –namely 20, 40 and 150 displacements per atom. Specifically, we investigate the evolution of the structural features, the mechanical properties, and the response to impact loading of Al_2_O_3_ thin films. Initially, the thin films contain a homogeneous dispersion of nanocrystals in an amorphous matrix. Irradiation induces crystallization of the amorphous phase, followed by grain growth. Crystallization brings along an enhancement of hardness, while grain growth induces softening according to the Hall-Petch effect. During grain growth, the excess mechanical energy is dissipated by twinning. The main energy dissipation mechanisms available upon impact loading are lattice plasticity and localized amorphization. These mechanisms are available in the irradiated material, but not in the as-deposited films.

Next generation nuclear systems, including advanced Light Water Reactors (LWRs), Generation IV concepts and fusion systems, will operate at higher temperatures and efficiencies than current generation LWRs. Improvements are also expected in regards to non-proliferation, fuel cycle efficiency, radioactive waste management, and safety^1^,^2^,^3^. However, the successful development of suitable materials for next generation nuclear systems continues to be a major challenge^4^. Most of the difficulties are related to the intense radiation fields to which materials will be exposed during operation. Radiation damage will exacerbate the most common failure mechanisms of engineering systems (i.e. thermal creep, fatigue, etc.), and will bring about additional degradation modes, such as irradiation creep, void swelling or high-temperature helium embrittlement, among others^5^,^6^,^7^. Further issues are related to the high operating temperatures that are necessary to increase power conversion efficiencies. In order to access the high temperature range (i.e. between 400 °C–1000 °C), coolants other than water must be utilized. The most promising coolants in this regard include supercritical water, high and very high temperature helium gas, molten salts, and liquid metals like sodium, lead, and lead-bismuth or lead-lithium eutectics. These coolants are also expected to improve neutronics and nuclear reactor physics. On the other hand, most of these coolants are extremely corrosive and detrimental to the reliability of in-core components^7^, and their inherently corrosive effects are augmented by high temperatures and radiation damage^8^.

In this context, ceramics represent a promising class of materials due to their high temperature strength, and due to their chemical inertness in several corrosive environments. The main issue with monolithic ceramics is their lack of plasticity at low homologous temperatures. A direct implication is that monolithic ceramics are inherently brittle. This fact excludes the utilization of monolithic ceramics as structural materials whenever catastrophic failure is not an option. Even though extrinsic toughening is an appealing route to overcome brittleness^9^, the widespread utilization of ceramics for structural components is difficult. On the other hand, the deposition of ceramic coatings on metallic structural materials can provide corrosion resistance without affecting structural requirements. It is worth highlighting that protective coatings are already being considered for accident tolerant fuel cladding for LWRs^10^,^11^. In the case of Generation IV concepts and fusion systems, coatings could be used to mitigate high temperature corrosion^7^,^12^ and tritium permeation^13^.

The dimensional changes induced by thermal creep, radiation damage, and their synergistic interactions can produce large stresses and strain at the coating-substrate interface. Therefore, an ideal coating must be able to accommodate such conditions while retaining both adhesion and structural integrity. For this reason, the mechanical performance of a given ceramic coating is extremely important. Room temperature processing would be an asset, in order to avoid microstructural changes in the substrate material. Further, in order to ensure the longevity of structures, an ideal coating must also be designed to withstand unparalleled radiation damage levels. On account of their fine grain size, *nanoceramic* coatings may benefit from the strength and chemical inertness of ceramics, combined with many favorable deformation modes^14^,^15^,^16^ and with the generally high radiation tolerance demonstrated in nanomaterials^17^,^18^,^19^,^20^,^21^,^22^,^23^,^24^.

Oxide nanoceramics in particular are a promising class of coating materials owing to their compatibility with a wide range of environments, including light water, liquid metals and high temperature helium^7^,^10^,^11^,^12^. The structural response of oxide nanoceramics to irradiation is usually characterized by an increase of the average grain size, as demonstrated for irradiated ceria thin films^25^,^26^,^27^, zirconia thin films^27^ and bulk yttria-stabilized zirconia^28^. However, the mechanical response of irradiated oxide nanoceramics remains largely unexplored. In this work, we report the evolution of the nanometer-scale structural features and the mechanical response of Al_2_O_3_ nanoceramic thin films as a function of radiation-induced grain growth. The damage exposures attained are similar to 20, 40 and 150 displacements per atom (dpa) at 600 °C. Such high levels of radiation damage approach or even exceed the damage exposures anticipated for next generation nuclear systems. Importantly, thin films are convenient model systems and are often used to study the fundamental effects of radiation on bulk nanocrystalline materials. The results obtained are expected to be similar in the case of bulk materials, provided that the pristine microstructural features are comparable.

## Methods

### Materials

Thin films of Al_2_O_3_ 1.3 μm thick are grown by Pulsed Laser Deposition (PLD) at room temperature on substrates of austenitic steel. Details of the PLD process are discussed elsewhere^29^. The substrate composition is 15Cr-15Ni-1.50Mo-1.5Mn-0.90Si-0.4Ti-0.09C-balance Fe (wt.%), and the sample size is 10 mm × 30 mm × 10 mm. This steel is generally representative of many austenitic steels being proposed for next generation nuclear reactors. Prior to Al_2_O_3_ thin film depositions, the substrates are ground, polished, sonicated, rinsed and then coated with a 200 nm FeCrAlY buffer layer (16.9Cr-6.4Al-0.1Y- bal. Fe - wt.%). The deposition of the buffer layer is conducted at room temperature using a radio-frequency sputtering source operated at 5 W/cm^2^ in an Ar environment (5 Pa, base pressure of 1.5.10^−3^ Pa), with an incidence angle of 45° and a target-to-substrate distance of 70 mm.

### Ion irradiations

In order to study the damage exposures anticipated for next generation nuclear systems in a short time and without facing activation issues, samples are irradiated with heavy ions as a surrogate technique of neutron irradiation. It is well-established that a reasonable equivalence in damage caused by ion or neutron irradiation can be achieved, provided that appropriate ion experimental parameters are selected^5^.

In this study, the ion energies are chosen to provide an irradiation range beyond the thickness of the thin films (deep into the substrate), in order to avoid chemical and interstitial injection effects (see Supplementary Information). Sets of four samples are irradiated at the JANNUS-Saclay platform^30^. Two ion beams are employed simultaneously to maximize radiation damage within the available beam time. The ion beams used are 12 MeV Au^5+^ ions and 18 MeV W^8+^ ions. The angle of incidence is 15° for both beams, and the total irradiated area is 3.14 cm^2^ (≈0.78 cm^2^ for each sample). Irradiations are carried out in vacuum (10^−5^ Pa) at 600 °C. This temperature is in the typical range of operating temperatures for Generation IV reactors. The temperature is continuously monitored, and is held constant throughout the experiment. The ion fluences are calculated by integrating the ion current measured by Faraday cups that are periodically interposed between the ion beams and the samples. In order to obtain results consistent with the model employed in the SPECTER code (used to calculate dpa levels from neutron irradiations), the damage exposures are calculated from ion fluences with the Stopping Range of Ions in Matter (SRIM) software^31^ according to the procedure described by Stoller *et al*.^32^. The damage levels reached within the thin films are 20 dpa (1.20 10^16^ W^8+^cm^−2^ + 1.74 10^16^ Au^5+^cm^−2^), 40 dpa (2.13 10^16^ W^8+^cm^−2^ + 2.76 10^16^ Au^5+^cm^−2^) and 150 dpa (4.52 10^16^ W^8+^cm^−2^ + 1.2 10^17^ Au^5+^cm^−2^). The peak damage for all the irradiations is always in the steel substrate, and exceeds 60 dpa, 120 dpa and 480 dpa in the three irradiations, respectively. The radiation damage at the coating-substrate interface is given by a marked gradient between the damage levels reached within the films and the peak damage levels. Importantly, the procedure described by Stoller *et al*.^32^ relies on quick damage calculations. We note that most of the investigations on irradiated oxide nanoceramics report full-cascade calculations, which yield damage levels that are higher by a factor of roughly 2. Therefore, the dpa levels reported in this study are not directly comparable to most of the studies on oxide nanoceramics, and should be multiplied by a factor around 2 before a direct comparison is made.

### Electron microscopy observations

Samples for Transmission Electron Microscopy (TEM) and Annular Dark Field-Scanning TEM (ADF-STEM) analyses are fabricated by sectioning a thin lamella by the conventional Focused Ion Beam (FIB) technique. The last step of the sample preparation involves low energy (2 KV) low current (100 pA) polishing of the lamella at ±6° (with respect to the ion beam) in order to minimize any damage artifacts from previous steps. TEM and ADF-STEM observations are performed with a FEI Titan (S)TEM. The predominantly diffraction contrast image formation of ADF is preferred over the mass contrast dominated imaging provided by high-angle (HA)-ADF imaging, as it provides for more clear images of the grain boundaries (GBs) in the nanoceramics. For each damage level, the grain size is averaged from the length of 50 grains using the line-intercept method along two directions, arbitrarily taken as along and orthogonal to the direction of the maximum length of each grain.

### Mechanical performance analyses

Quasi-static load-controlled indentations are performed at room temperature using a Micromaterials Nanotest system equipped with a Berkovich diamond tip. The indentations are applied in multiple arrays of nine indentations each. The maximum load is 10 mN, corresponding to penetration depths below one tenth of the thickness of the Al_2_O_3_ films. The maximum penetration depth is 120 ± 3 nm for the as-deposited thin films, and 97 ± 3 nm, 99 ± 3 nm and 97 ± 8 nm for the thin films irradiated up to 20 dpa, 40 dpa and 150 dpa, respectively. In all the tests, the maximum load is held constant for 5 seconds, in order to allow creep strain to saturate. A 60 second holding is set at 20% of the unloading curve to measure thermal drift. Machine compliance and thermal drift are taken into account by assuming a constant rate throughout the test. The mechanical properties of the material (e.g. the Young’s modulus *E* and hardness *H*) are assessed from the load-displacement curves following the Oliver and Pharr approach. *E* is computed from the reduced Young’s modulus *E*_*r*_ using the formula 1/*E*_*r*_ = (1 − ν^2^)/*E* + (1 − ν_*d*_^2^)/*E*_*d*_, where ν, ν_*d*_, and *E*_*d*_ are the Poisson’s ratio of the material, and the Poisson’s ratio and the Young’s modulus of the diamond tip, respectively. The Young’s modulus *E* is deduced from *E*_r_ assuming that *ν* decreases following an inversely proportional relationship with *E*_r_ (due to crystallization and grain growth) -for instance, from the reported value of 0.29 at 0 dpa^29^, to 0.27 at 20 dpa, 0.26 at 40 dpa, and 0.23 at 150 dpa. Note that variations of ±10% on the assumptions of ν yield variations of *E* of approximately 5%.

The response of the thin films to impact loading is evaluated using nanoimpact tests. For these tests, a Micromaterials Nanotest system is used to blast a cube-corner diamond tip periodically towards the surface of the samples. The tip is blasted on the samples’ surface from a fixed distance (10 μm) with a well-defined force (1 mN) through a solenoid behind the tip that is connected to a time relay. The corresponding speed of impact is 500 μm/s. For each impact cycle, the force is held for 3 seconds while displacement is recorded, and it is then released over the next 2 seconds. Three sets of 10 impact cycles are performed for each damage exposure.

## Results and Discussion

**Figure 1:**
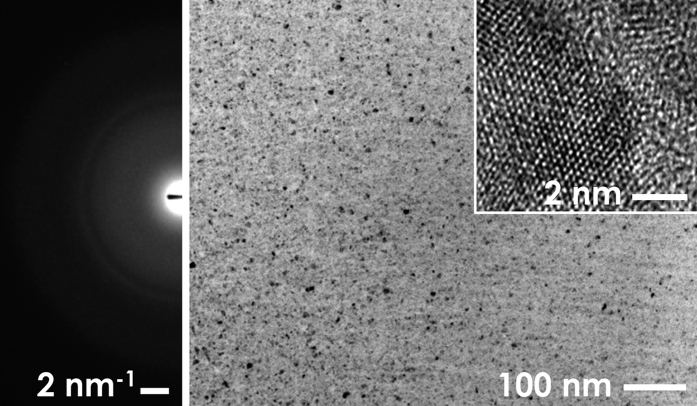
BF-TEM micrograph, and high-resolution (HR) close-up (inset) of the nanostructure of the as-deposited Al_2_O_3_ thin films showing a homogeneous dispersion of a low volume fraction of randomly-oriented nanocrystalline Al_2_O_3_ domains in an amorphous Al_2_O_3_ matrix.

The Bright-Field TEM micrographs shown in Fig. 1 display the nanostructure of the as-deposited Al_2_O_3_ thin films. The dark contrast spots correspond to randomly-oriented ultra-fine nanocrystalline γ-Al_2_O_3_ domains (6 ± 4 nm), whereas the bright contrast results from the presence of the amorphous phase of Al_2_O_3_. The appearance of a relatively sharp ring, together with a diffused intensity halo in the diffraction pattern (DP) confirms that the the material consists of a dual phase structure, with the amorphous phase dominating the overall structure. The volume fraction of γ-Al_2_O_3_ nanocrystalline domains is very low at approximately 1% (see Supplementary Information).

The main advantage of this type of dual structure over a fully nanocrystalline structure is that it confers an unusual ensemble of metal-like mechanical properties (Young’s modulus *E* = 195 ± 9 GPa, ν = 0,29 ± 0,02) and moderate hardness (*H* = 10 ± 1 GPa) to the Al_2_O_3_ thin films. In particular, the amorphous matrix precludes grain sliding, enables plastic deformation and inhibits crack nucleation^29^. Despite the lower hardness compared to single crystal sapphire (*H*_*sapphire*_ = 27.6 ± 2 GPa)^33^, the Al_2_O_3_ thin films are still significantly harder than most metallic materials. Moreover, the resulting *H*/*E* ratio (i.e. 0.051) is comparable with the *H*/*E* ratios of superhard nanocomposite coatings for tribological applications^34^. This may be beneficial for example during fuel rod insertion or grid-to-rod fretting during operation.

**Figure 2:**
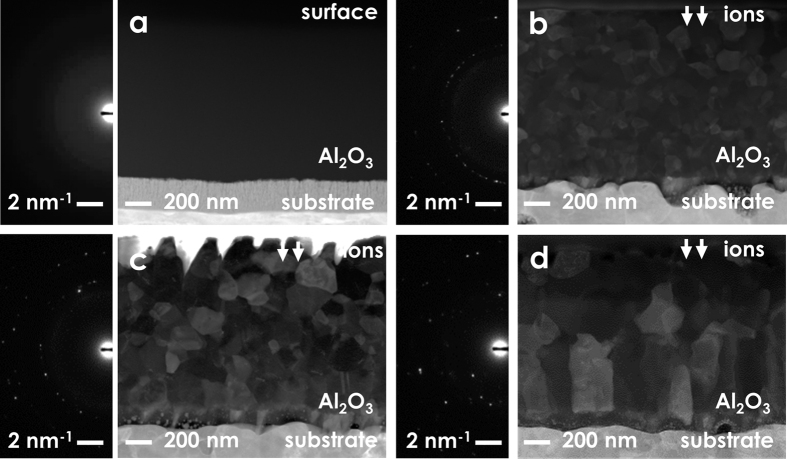
ADF-STEM micrographs and DPs showing as-deposited (**a**) and irradiated Al_2_O_3_ thin films after 20 dpa (**b**), 40 dpa (**c**) and 150 dpa (**d**) at 600 °C. The coarsening induced by irradiation releases excess free energy due to the interaction between point defects and GBs^26^.

The ADF-STEM micrographs in Fig. 2 show the structural features of the as-deposited and the irradiated thin films. These images indicate that a fully nanocrystalline structure is realized upon irradiation, and that extended irradiations induce grain growth as the dpa levels are increased. The average grain size increases from 6 ± 4 nm to 101 ± 56 nm at 20 dpa, 153 ± 62 nm at 40 dpa and 293 ± 85 nm at 150 dpa (Fig. 2b–d). The crystallization and grain growth observed manifest as an evolution of the DPs from a diffused intensity halo to rings and isolated spots. The crystalline phases present in the irradiated nanoceramic are γ-Al_2_O_3_ up to 40 dpa, and both γ-Al_2_O_3_ and α-Al_2_O_3_ at 150 dpa (see Supplementary Information). It is worth highlighting that the irradiation did not induce any loss of adhesion or delamination effects at the thin film-substrate interface. The combined effect of irradiation and strain imposed by the substrate (for instance, due to thermal and irradiation creep or swelling) is beyond the scope of this study, and warrants further investigation.

**Figure 3:**
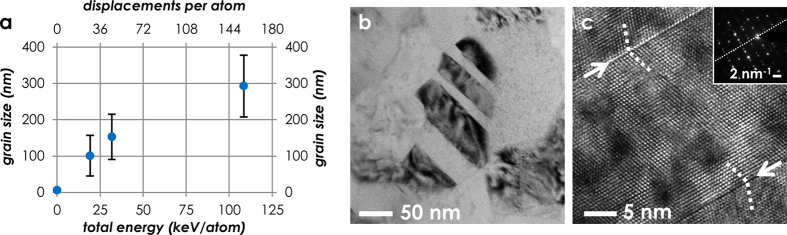
Grain growth in the Al_2_O_3_ thin films as a function of total energy injection and displacive radiation damage (**a**). The grain coarsening is accompanied by the formation of twin boundaries (**b**), which release accumulated mechanical energy. The presence of a mirror plane in both the HR-TEM micrograph (**c**) (indicated by arrows), and in the DP inset confirms the twin relationship of the adjacent grains.

It is likely that temperature plays an important role in determining the kinetics of the structural evolution. However, we attribute crystallization and grain growth to the sole effect of irradiation (see Supplementary Information). The initial crystallization is expected to occur readily upon irradiation, and may be homogeneous^35^, epitaxial^36^,^37^, or both. The subsequent coarsening effect can be explained in terms of a fast disorder-driven mechanism, which is available even below room temperature^25^,^26^, and which is governed by the capture of interstitials by GBs^22^,^23^,^24^. The incident ions introduce a large amount of local disorder through atomic displacement cascades. The disordered regions interact with GBs, releasing excess free energy and leading to an overall growth. It is also interesting to notice that the extent of grain growth is strongly influenced by the total amount of energy injected by the ions into the material^27^. In the energy range investigated, the energy of the ions is transferred to the material both by electronic excitations and displacive damage (i.e. nuclear collisions). The effect of these different kinds of energy loss may be additive, synergistic or even competing. In the case of oxide nanoceramics, the effect is generally additive^27^. The plot in Fig. 3a shows the dependence of grain growth both on the total amount of energy injected into the material (keV per target atom), and on displacive radiation damage (displacements per atom). The graph indicates that radiation-induced grain growth is a self-limiting process, which follows a sublinear dependence on damage exposure, in good agreement with previous results concerning other nanocrystalline oxides^25^,^26^,^27^,^28^.

In the irradiated material, grain growth is accompanied by the formation of planar defects with two parallel flat boundaries. These defects are found occasionally, and their presence is independent of damage exposure. The defects are identified as twins, and an example is shown in Fig. 3b. The presence of a mirror-plane both in the high-resolution TEM (HR-TEM) micrograph and in the DP inset in Fig. 3c confirms that the defects observed are indeed twins. The formation of twins in nanocrystalline solids can be understood in terms of mechanisms such as nanoscale multiplane shear^38^ or stacking fault formation led by Shockley partial dislocations^39^. From an energy balance perspective, the formation of twins may be explained by the need to release the excess mechanical energy accumulated during incoherent grain coarsening.

**Figure 4:**
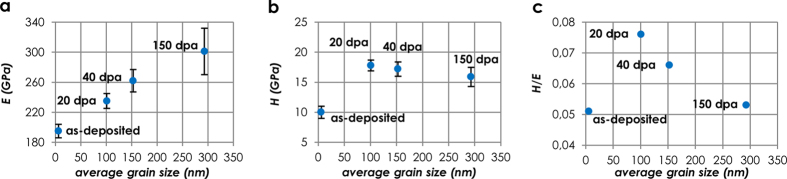
Effect of radiation-induced grain growth on the mechanical properties of Al_2_O_3_ nanoceramic thin films, namely the Young’s modulus *E* (**a**), the hardness *H* (**b**) and the hardness to Young’s modulus ratio *H*/*E* (**c**). The trend of hardness is well-described by the Hall-Petch effect, due to the increase of grain size with increasing damage exposures.

The structural rearrangements induced by the irradiations (i.e., crystallization and grain growth) bring about changes in the mechanical properties of the material. These changes are plotted in Fig. 4 as a function of the average grain size. The reduced Young’s modulus *E*_*r*_ increases monotonically with grain size (i.e. *E*_*r,20dpa*_ = 205 ± 7 GPa, *E*_*r,40dpa*_ = 222 ± 10 GPa, and *E*_*r,150dpa*_ = 245 ± 19 GPa). Accordingly, the Young’s modulus *E* increases from *E*_*20dpa*_ = 235 ± 10 GPa, to *E*_*40dpa*_ = 262 ± 15 GPa and *E*_*150dpa*_ = 301 ± 31 GPa (Fig. 4a). The hardness *H* (Fig. 4b) peaks at moderate damage exposures, varying from *H*_*20dpa*_ = 17.8 ± 0.9 GPa, to *H*_*40dpa*_ = 17.2 ± 1.2 GPa, and *H*_*150dpa*_ = 15.9 ± 1.6 GPa. Notably, the trend is well described by the Hall-Petch effect, whereby a material’s strength and hardness decrease as the average grain size increases. The Hall-Petch relationship describes the measured hardness *H*_*v*_ according to the formula *H*_*v*_ = *H*_*0*_ + *kD*^−1/2^, where *H*_*0*_ is the intrinsic hardness dependent on frictional lattice resistance to dislocation motion, *k* is the material-specific strengthening coefficient, and *D* is the average grain size. In this work, the best linear fit of *H*_*v*_ as a function of *D*^−1/2^ yields *H*_*0*_ = 13.255 GPa and *k* = 46.638 GPa.nm^1/2^, with a coefficient of determination equal to *R*^2^ = 0.9756. Below the so-called *strongest grain size* (typically in the range 10–20 nm^40^), the strengthening effect is balanced by GB shear, which yields a reduction of hardness for decreasing grain size. This effect is usually referred to as the inverse Hall-Petch effect. A detailed overview of the mechanisms that yield an enhancement of hardness in nanoceramics has been recently reported by Veprek^40^.

A direct comparison between the mechanical properties of the irradiated thin films and polycrystalline α-Al_2_O_3_ is difficult. The mechanical properties of the latter vary depending on the grain size, the presence of impurities and on the processing route^41^. However, comparisons can be made with bulk nanocrystalline α-Al_2_O_3_ (bnc-alumina). The reported hardness and stiffness for bnc-alumina with a grain size of 150 nm are *H*_*bnc-alumina*_ = 25.5 ± 0.3 GPa and *E*_*bnc-alumina*_ = 403 GPa^42^,^43^. Here, the maximum hardness is reached when the average grain size is 101 nm (*H*_*20dpa*_ = 17.8 ± 0.9 GPa). The corresponding Young’s modulus is *E*_*20dpa*_ = 235 ± 10 GPa. The differences observed are probably due to: (i) the presence of different phases (γ-Al_2_O_3_ versus α-Al_2_O_3_), (ii) the measurement method (Berkovich nanoindentation versus Vickers microindentation), (iii) the presence of radiation-induced point defects, or (iv) combinations thereof.

Another important implication of the observed irradiation-induced crystallization is that the *H*/*E* ratio of the thin films is enhanced in response to irradiation. The *H*/*E* ratio peaks at moderate damage exposures (when the volume fraction of GBs is the highest), varying from 0.051 for the as-deposited condition, to 0.076, 0.066 and 0.053 for 20, 40 and 150 dpa, respectively (Fig. 4c). These results suggest an improvement in service of the robustness of the thin films against wear. This is of particular interest concerning liquid metal erosion^44^ or rod-to-grid fretting^45^.

Fracture toughness of ceramic materials is typically determined from nanoindentation tests by measuring the length of surface radial cracks emanating from the corner of imprints. This type of measurement is not possible here because cracks are not observed in any case, due to the low load and the low film thickness. However, an indirect estimation of fracture toughness is given by the *H*/*E* ratio^34^. The as-deposited material lacks long-range order and nanostructural defects (such as dislocations) that may shield stress and suppress crack openings. Thus, the attainable plasticity in the wake of a crack tip is limited, and any opening would be likely accommodated by unstable crack propagation. Accordingly, the *H*/*E* ratio of the pristine material is comparatively low. In contrast, the mechanical response of the irradiated material is mainly driven by GBs. The large volume fraction of GBs makes new energy dissipation mechanisms available (e.g., twinning). The resulting *H*/*E* ratio is higher, which suggests an enhancement of fracture toughness. Although thin film coatings are not structural components, an enhancement in service of fracture toughness is desirable because future design rules might rely entirely on the presence of a coating for the correct operation of a reactor. In this perspective, a certain extent of cracking may be acceptable during the extended exposure to neutron radiation fields, while unstable crack propagation would certainly not be an option.

**Figure 5:**
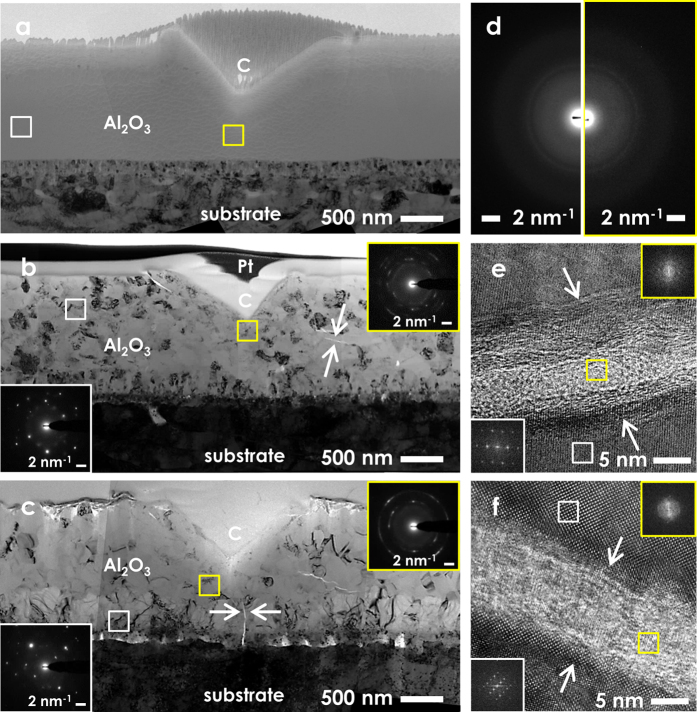
Cross-sectional TEM micrographs of representative nanoimpact imprints on the Al_2_O_3_ nanoceramic thin films before (**a**) and after irradiation up to 20 dpa (**b**) and 150 dpa (**c**). No major structural rearrangements are induced by impact loading in the unirradiated samples, as confirmed by the identical SADPs gathered distant from and below the impact imprint (**d**). The appearance of arcs and rings in the SADPs beneath the impact zones in the irradiated samples is due to energy dissipation through bending of the lattice planes. Another energy dissipation mode present is localized amorphization, which is indicated by arrows in (**b**,**c**), and shown in high-resolution in (**e**,**f**). The FFT insets in (**e,f**) confirm that the bright contrast corresponds to the amorphous phase, and that the dark contrast corresponds to the crystalline phase.

Additional qualitative evidence in support of the enhancement of fracture toughness is provided experimentally by nanoimpact tests. In these tests, a cube-corner diamond tip is periodically blasted against the surface of the thin films. The impact depth is the highest for the as-deposited thin films (see Supplementary Information), which suggests that the impact energy is dissipated more efficiently in the irradiated samples. As a matter of fact, the impact response of the as-deposited and the irradiated thin films is radically different. Figure 5 displays the cross-sectional images of representative nanoimpact imprints for as-deposited and irradiated samples. Two selected area (SA) DPs are acquired for each cross-section, both distant from (white box) and within (yellow box) the impact zone (below and in the vicinity of the impact imprint). In the unirradiated samples (Fig. 5a), impact energy is dissipated through shear banding, and no major structural rearrangements are induced by the impact loading. This observation is confirmed by the fact that the SADPs gathered distant from and within the impact imprint appear identical, as shown in Fig. 5d. Figure 5b,c show the cross-section of nanoimpact imprints in samples exposed to 20 dpa (corresponding to the peaks of *H* and *H/E* ratio in Fig. 4b,c) and 150 dpa (end-of-life exposure), respectively.

The appearance of arcs and rings in the SADPs beneath the impact zones is due to the bending of lattice planes, which denotes plastic strain as one of the main energy dissipation mechanisms. Another energy dissipation mechanism present is localized amorphization. Notably, crystalline-to-amorphous phase transitions are often described as toughening mechanisms^9^. The HR-TEM micrographs in Fig. 5e,f show the localized amorphization indicated by arrows in Fig. 5b,c, respectively. The FFT insets show a diffused halo where the contrast is bright, and diffraction spots where the contrast is dark, confirming the amorphous nature of the bright contrast band, and the long-range order of the contiguous zone. The close alignment of the crystal lattice on either side of the amorphous band rules out the formation and subsequent rebonding of two cracked surfaces. Localized amorphization has been observed in sapphire^46^ and in other unirradiated ceramics exposed to shock loading, such as B_4_C^47^,^48^, SiO_2_^49^, Y_2_Si_2_O_7_^50^ or B_6_O^51^. The onset of the phenomenon has been explained by shock-induced plastic waves^46^ and shear instability^48^,^49^. These phenomena are relevant at the extremely high stresses and strain rates induced by shock loading. Further causes include the coalescence of dislocation loops under high shear stresses, as occurs upon quasi-static mechanical load^50^,^51^, and adiabatic shear, which is governed by elastic strain energy in brittle solids^52^. Arguably, both the high strain rate induced by impact loading, and the coalescence of defects and defect clusters formed during irradiation may play an important role in the amorphization process observed here. However, the impact speed in this study (≈500 μm/s) is several orders of magnitude lower than the impact speed in shock loading experiments (≈18 km/s)^46^,^47^,^48^,^49^. This fact suggests that shock-induced plastic waves and shear instability are unlikely as the main driving forces.

It is worth noting that the utility of oxide nanoceramics as radiation tolerant materials is often thought to be limited by grain growth. Indeed, GBs are usually considered as the actual source of *radiation tolerance* due to their efficient behavior as defect sinks. The problem is that the density of GBs decreases inexorably as grain growth proceeds. Thus, the radiation tolerance of oxide nanoceramics is expected to fade away for increasing radiation damage exposures. However, radiation tolerance can be defined in many ways. For example, radiation tolerance can also be conceived in terms of the expected lifecycle of a given component. From this point of view, nanoceramic thin films can be utilized as radiation tolerant coatings indeed. As a matter of fact, the thin films in this work are able to withstand radiation damage up to 150 dpa without suffering catastrophic failure nor delamination. It is also worth noting here that coarse-grained polycrystalline α-Al_2_O_3_ suffers void swelling and releases the resulting stresses through cracking at much lower damage exposures^53^,^54^.

To conclude, the results of this work may be extended to other amorphous or nanoceramic oxides in bulk or thin film form, or even to other compounds. First, it is well-established that radiation-induced crystallization occurs in a range of amorphous compounds, and not only in Al_2_O_3_. For example, radiation-induced crystallization has been reported for InP and InAs seminconductors^55^ and MgAl_2_O_4_ spinel^56^. The onset of crystallization in such compounds (and perhaps in Al_2_O_3_, too) is given by a tradeoff between temperature, dose and dose rate^55^,^56^. Second, the transformation of an amorphous compound into a nanocrystalline material should always make new energy dissipation mechanisms available, such as twinning or grain rotation, among others. The specific mechanisms are likely material-dependent. Third, radiation-induced grain growth is commonly observed in bulk oxide nanoceramics, in ceramics other than oxides, and in metals^57^. Since the Hall-Petch relationship describes a fundamental effect that holds for a wide variety of materials, we expect that grain growth should usually be accompanied by an initial increase of hardness according to the inverse Hall-Petch effect. This stage should be followed by Hall-Petch softening for grain sizes above the strongest grain size. Because the strongest grain size is usually rather low (in the range 10–20 nm), it is reasonable to expect a Hall-Petch softening effect already at low radiation damage levels. This effect may be influenced by the presence of radiation-induced point defects and defect clusters, depending on the material under consideration. Last, but not least, the effect of high radiation damage levels may vary conspicuously depending on the class of material considered. Thus, it seems hard to extrapolate our findings to other classes of materials exposed to extended irradiations.

## Conclusions

In this work, amorphous/nanocrystalline Al_2_O_3_ thin films are deposited on austenitic steel substrates. The thin films are irradiated with heavy ions up to 20 dpa, 40 dpa and 150 dpa at 600 °C. Initially, irradiation induces an amorphous-to-crystalline transformation resulting in a fully nanograined structure, while extended irradiations induce grain growth and softening in accordance with the Hall-Petch relationship. The Young’s modulus of the thin films increases monotonically with increasing dose, while the *H*/*E* ratio increases upon crystallization and decreases thereafter, yet with a final value which is still higher than the initial value. The initial increase of the *H*/*E* ratio suggests a potential improvement in the fracture toughness of the irradiated thin films. The improvement seems to be manifest in the onset of such energy dissipation mechanisms as twinning (during grain growth), and lattice plasticity and localized crystalline-to-amorphous transformations (under impact loading). These energy dissipation mechanisms are present at both extremes of the damage levels studied.

Overall, the findings in this work encourage the use of nanoceramics in radiation environments well beyond the traditional limiting range for standard nuclear materials. In particular, the results lend support for the use of nanoceramic coatings for in-core, high radiation field components with enhanced corrosion and wear resistance, and possibly even their use in bulk form. Straightforward applications include accident tolerant fuel concepts for advanced light water reactors, fuel cladding for Generation IV systems, and tritium breeding components for fusion tokamaks.

## Additional Information

**How to cite this article**: García Ferré, F. *et al*. Radiation endurance in Al_2_O_3_ nanoceramics. *Sci. Rep.* **6**, 33478; doi: 10.1038/srep33478 (2016).

## Supplementary Material

Supplementary Information
