# Supplementary material for: Radiation endurance in Al2O3 nanoceramics
Source: Sci Rep. 2016 Sep 22;6:33478. doi: 10.1038/srep33478 (PMC5031969; doi:10.1038/srep33478)
Supplement: Supplementary Information [file srep33478-s1.pdf]

Supplementary Information

Radiation endurance in Al_2O_3 nanoceramics

F. García Ferré[1,†], A. Mairov[2,†], L. Ceseracciu[3], Y. Serruys[4], P. Trocellier[4], C. Baumier[5], O. Kaïtasov[5], R. Brescia[6], D. Gastaldi[7], P. Vena[7], M.G. Beghi[8], L. Beck[4], K. Sridharan[2] and F. Di Fonzo[1,*]

1. Center for Nano Science and Technology @PoliMi, Istituto Italiano di Tecnologia, Via Pascoli 70/3, 20133 Milano (MI), Italia.

2. Department of Engineering Physics, University of Wisconsin-Madison, 1500 Engineering Drive, 53715 Wisconsin (WI), USA.

3. Smart Materials, Nanophysics, Istituto Italiano di Tecnologia, Via Morego 30, 16163 Genova (GE), Italia.

4. Service de Recherches de Métallurgie Physique, Laboratoire JANNUS, CEA, DEN, F-91191 Gif-Sur-Yvette, France.

5. CNRS/IN2P3/CSNSM/SEMIRAMIS/JANNUS-Orsay, Université Paris Sud, Bat. 108, 91400 Orsay, France.

6. Department of Nanochemistry, Istituto Italiano di Tecnologia, Via Morego 30, 16163 Genova (GE), Italia.

7. Dipartimento di Chimica, Materiali ed Ingegneria dei Materiali, Politecnico di Milano, Via Mancinelli 7, 20131 Milano (MI), Italia.

8. Dipartimento di Energia, Politecnico di Milano, Via Ponzio 34/3, 20133 Milano (MI), Italia.

[†]These authors contributed equally to the work.

[*]email: fabio.difonzo@iit.it

Content:

1. Ion implantation

2. Thermal stability

3. Phase evolution

4. Nanoimpact

1. Ion implantation

According to SRIM calculations, the implantation peaks for the 12 MeV Au^{5+} ions and the 18 MeV W^{8+} ions are beyond 1,6 μm and 2 μm from the surface of the samples, respectively. Figure S1 shows the implantation depth profiles for the 12 MeV^{5+} Au and 18 MeV W^{8+} ions as simulated by SRIM, together with the resulting radiation damage profile.

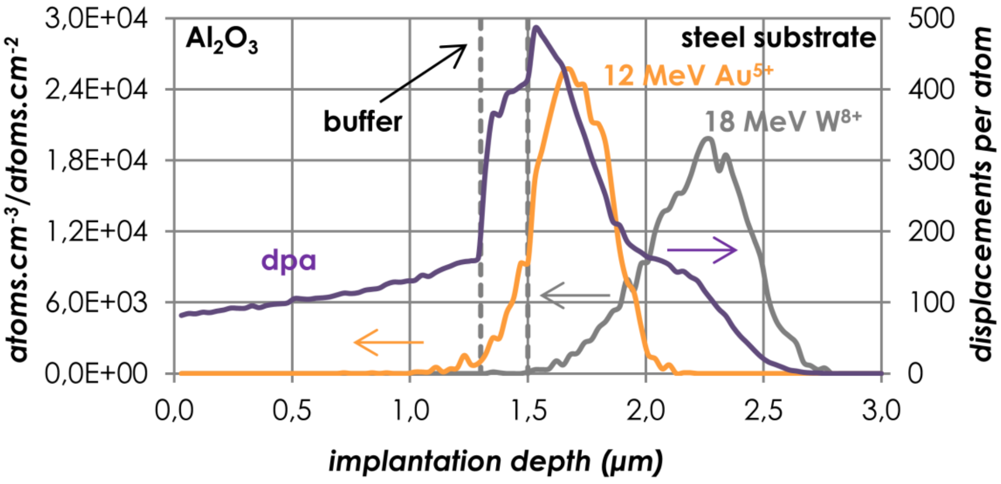

Figure S1. Implantation depth for 12 MeV Au^{5+} and 18 MeV W^{8+} ions, together with the resulting damage profile. Note that the ions are implanted beyond the Al_2O_3 thin films.

High-angle annular dark-field – scanning TEM (HAADF-STEM) observations coupled with energy-dispersive X-ray spectroscopy (EDS) confirm that the 12 MeV Au^{5+} ions are implanted beyond the

nanoceramic film and into the substrate, as shown in Figure S2. The 18 MeV W^{8+} ions are lighter and more energetic than gold ions. Therefore, these ions can be expected to be implanted beyond the nanoceramic. This statement cannot be verified directly with reasonable reliability by EDS mapping due to the superimposition of the relevant EDS peaks for W with the ones of other elements present in the sample (e.g. Au).

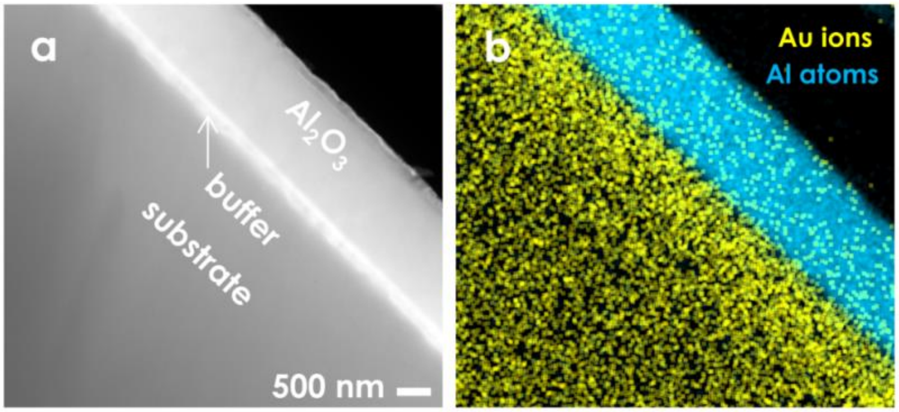

Figure S2. HAADF-STEM micrograph (a) and EDS map (b) showing that Au ions are implanted beyond the nanoceramic thin film. In (b), the implanted Au ions are shown in yellow, whereas the Al atoms of the Al_2O_3 thin films are shown in blue.

It is worth pointing out that because the ions are implanted beyond the thin films, the entire thickness of the oxide is affected by grain growth (as shown in figure 2 in the main text). In comparison, whenever the irradiated samples are bulk nanoceramics, grain growth is confined to a surface region. The depth of this region is determined by the range of the ions utilized. In some cases, stresses may build up at the irradiated-unirradiated interface, since the gradient of radiation damage in that zone is high and grain growth only occurs in the irradiated side of the interface. These stresses may induce intergranular cracking, as found by S. Dey and co-authors [28]. The same authors have claimed that a more uniform distribution of damage (such as within a nuclear reactor) would probably suppress

cracking. The absence of cracks in the present work (in which the distribution of damage is more uniform) seems to support such statement.

The elemental mapping shown in figure S2 is done with a JEOL JEM-2200FS TEM (with image C_S-corrector) equipped with a Bruker Quantax 400 system with a 60 mm^2 XFlash 6T silicon drift detector (SDD). For these analyses a cross-section sample is obtained by conventional preparation techniques on a sample irradiated up to 150 dpa. The reported maps are obtained by a routine of the Bruker Esprit software allowing for background subtraction, signal deconvolution and integration of the Al K_α, Au L_α and W L_α point by point in the image, needed due to partial superimposition of the signals of interest.

2. Thermal stability

In order to confirm that crystallization and grain growth are due to the sole effect of irradiation, samples are observed by TEM *in-situ* following annealing and ion irradiation at the JANNUS platform of CNRS-Orsay. The observations are done with a 200 kV Tecnai G20 TEM equipped with a custom polar piece, coupled to an ion accelerator. The experiment is designed according to the criteria described in Methods. Both the annealing and the irradiation are performed in vacuum (10^{-5} Pa) at 600°C, and the irradiation is done with 150 keV Ni^{2+} ions up to a fluence of $2.88.10^{16}$ ions/cm^2, which corresponds to roughly 25 dpa. The samples are prepared by depositing 30 nm thick Al_2O_3 films directly onto 200 μm thick silicon TEM grids with a 20 nm thick amorphous Si_3N_4 membrane support.

Figure S3a shows the pristine structure of the nanoceramic. The maximum grain size is below 12 nm. No discernible crystallization or grain growth are found during annealing for 90 minutes at 600°C (Fig. S3b). This observation is also confirmed by the fact that the DPs shown as insets remain unchanged. By contrast, irradiation at 600°C for 90 minutes induces crystallization and coarsening, as shown in figure S3c. The maximum grain size after irradiation exceeds 20 nm. The discretization and the

appearance of new rings in the DP inset of figure S3c provide further evidence of the onset of structural rearrangements following irradiation (i.e. crystallization and grain growth).

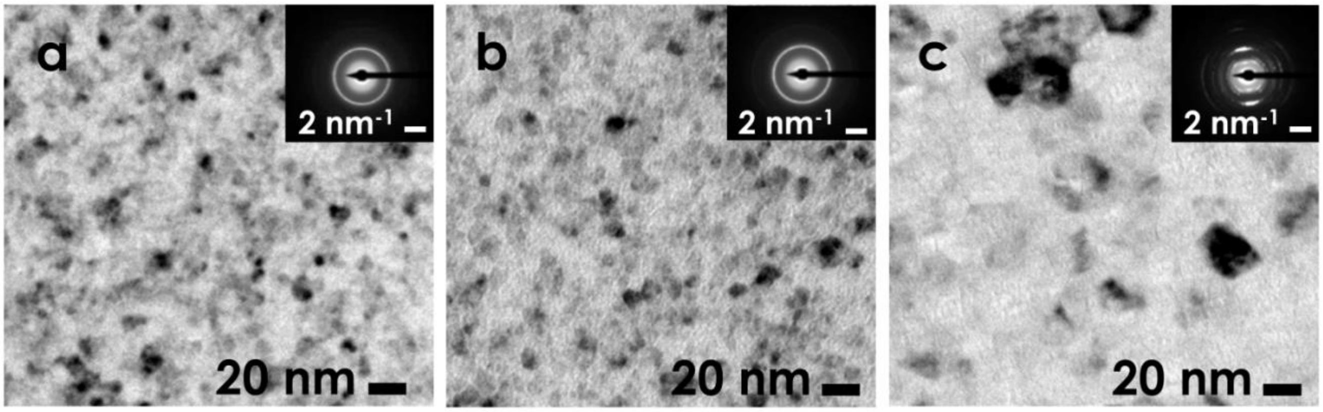

Figure S3. TEM micrographs and DP insets showing the structure of the nanoceramic in its pristine condition (a), after annealing for 90 minutes at 600°C (b), and after irradiation for 90 minutes (roughly 25 dpa) at 600°C (c). Crystallization and grain growth are observed only in the irradiated sample.

Conclusive evidence of the fact that crystallization and grain growth are only due to irradiation is provided by systematic observations of the masked region (e.g. an unirradiated region) of irradiated samples (i.e. samples described in the main text, irradiated with gold and tungsten ions). The TEM observations show that no crystallization occurs at the irradiation temperature (600 °C) for the entire duration of the experiments. Figure S4 shows a TEM micrograph of the unirradiated region of a sample exposed to 150 dpa. In this case, the duration of the irradiation is roughly 24 hours. The micrograph does not reveal any appreciable structural rearrangement, as also confirmed by the DP, which remains basically unchanged compared to the pristine DP (see figure 1 in the main text).

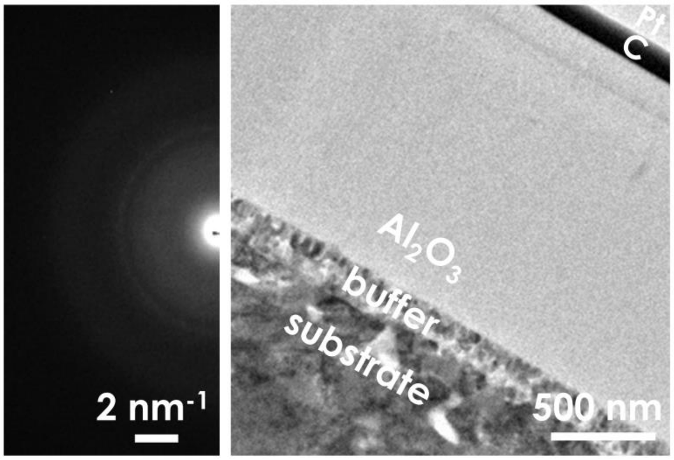

Figure S4. TEM micrograph and DP of an unirradiated region of a sample exposed to 150 dpa at 600°C. No structural rearrangements are caused by the thermal annealing in the nanoceramic.

3. Phase evolution

The crystalline phase present in the pristine material is analysed by DP indexing in samples grown directly onto TEM grids with an amorphous carbon support. The films are 50 nm thick. Figure S5 shows a dark field TEM (DF-TEM) micrograph of the structure of the pristine nanoceramic (Fig. S5.a), together with the corresponding DP and the azimuthally integrated profile (Fig. S5.b). The profile is compatible with γ-Al_2O_3 according to card ICSD 30267. Based on the DF-TEM micrograph shown (Fig. S5.a), the volume fraction of the γ-Al_2O_3 domains dispersed in the amorphous matrix is above 0.3378%. However, since not all the nanocrystalline domains diffract under the particular condition used for the micrograph (which is obtained by selecting just a fraction of the most intense rings in the DP, roughly 120° wide), the actual volume fraction is higher –at least by a factor of roughly 3. Thus, the actual volume fraction of the γ-Al_2O_3 domains is around 1%.

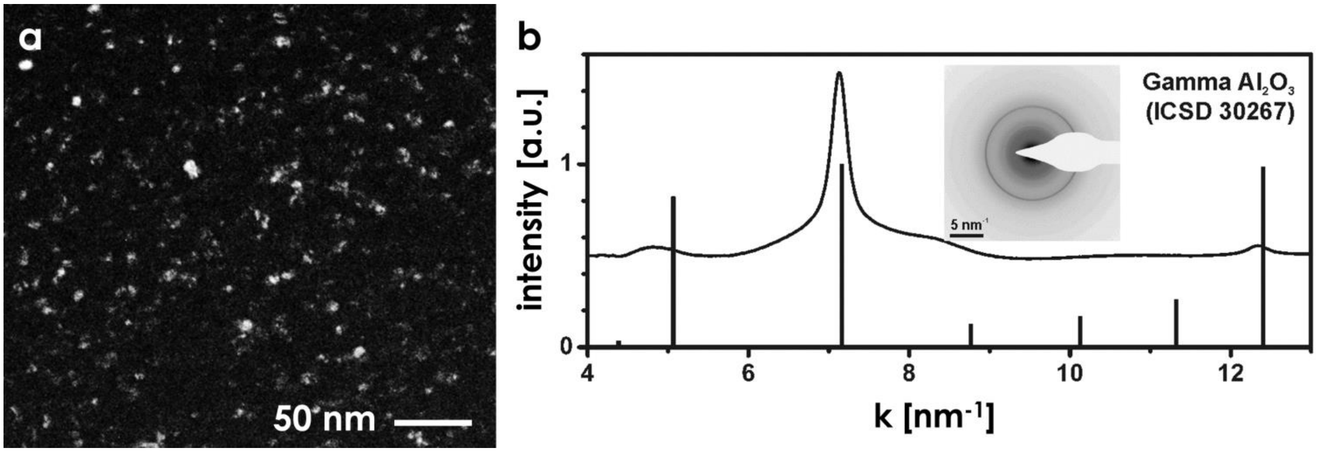

Figure S5. DF-TEM micrograph of the pristine nanoceramic (a). The azimuthally integrated DP profile (b) is compatible with γ-Al_2O_3.

The crystalline phases present in the irradiated nanoceramic are analyzed by X-Ray Diffraction (XRD). The data are obtained using a Bruker D8 X-ray diffractometer, with Cu-Kα radiation in the Bragg-Brentano geometry. Figure S6 displays the XRD spectra collected from pristine samples, and after 20 dpa, 40 dpa and 150 dpa. The as-deposited thin films are XRD-amorphous, and the only peaks present in the pristine samples belong to the austenitic steel substrates (card ICCD PDF 04-002-1864). The XRD spectra of the irradiated samples reveal the presence of γ-Al_2O_3 (card ICCD PDF 00-050-0741) independently from the radiation damage exposure. Only the XRD spectrum of samples exposed to 150 dpa reveals the presence of a new phase –namely, α-Al_2O_3 (card ICCD PDF 00-011-0661). Despite there seems to be a phase evolution upon high radiation damage levels, a systematic study is necessary to establish what the exact phase evolution is. Future investigations will focus on this point.

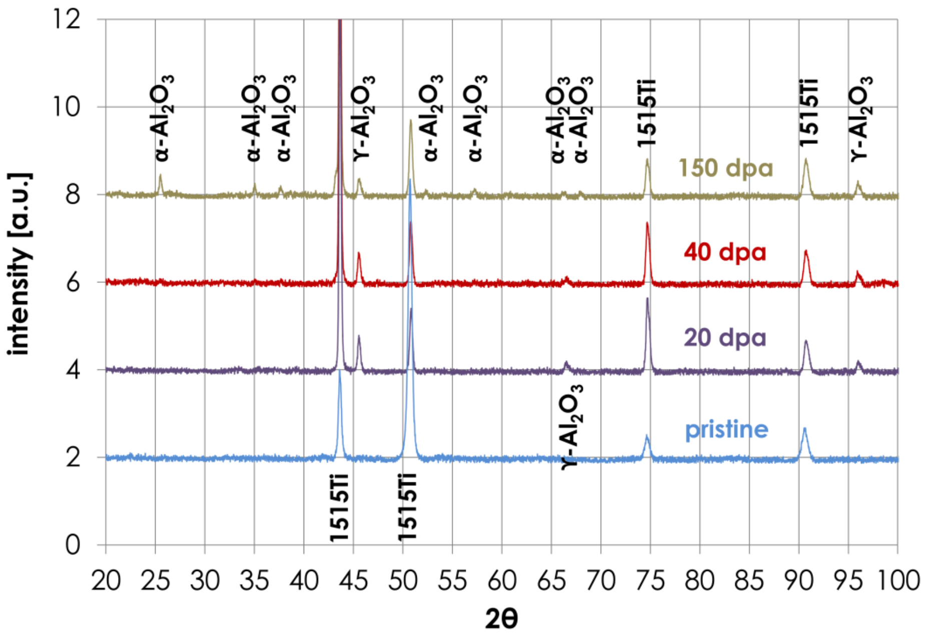

Figure S6. XRD spectra of the pristine and the irradiated Al2O3 thin films. The pristine material is XRD-amorphous. The irradiated samples always contain γ-Al_2O_3. Also α-Al_2O_3 is found in samples exposed to 150 dpa.

4. Nanoimpact

The nanoimpact tests performed in this study provide qualitative evidence on the enhancement in-service of the fracture toughness of the Al_2O_3 nanoceramic. The results of the tests are plotted in figure S7a in terms of impact depth as a function of the number of impacts. The impact depth is the highest for the as-deposited thin films, suggesting that the impact energy is dissipated more efficiently in the irradiated samples. The absence of sharp variations in all the nanoimpact curves indicates that no major failure takes place in any of the tested samples, as confirmed by SEM analyses of the nanoimpact zones (Fig. S7b). The evolution of impact depth is similar in all cases, meaning that similar mechanisms govern the impact response, and suggesting that the toughness is comparable. Anyhow, the penetration is larger in the pristine material, which can be attributed to the lower hardness only in part. In addition, the impact energy seems to be dissipated more effectively for samples irradiated up to 20 or 40 dpa, in good agreement with the trend for the *H/E* ratio (Fig. 4c). This fact is particularly clear during the first

impact. Note that saturation is reached after several impacts because the applied force is distributed over increasing contact areas. In other words, the contact pressure decreases as the number of impacts increases.

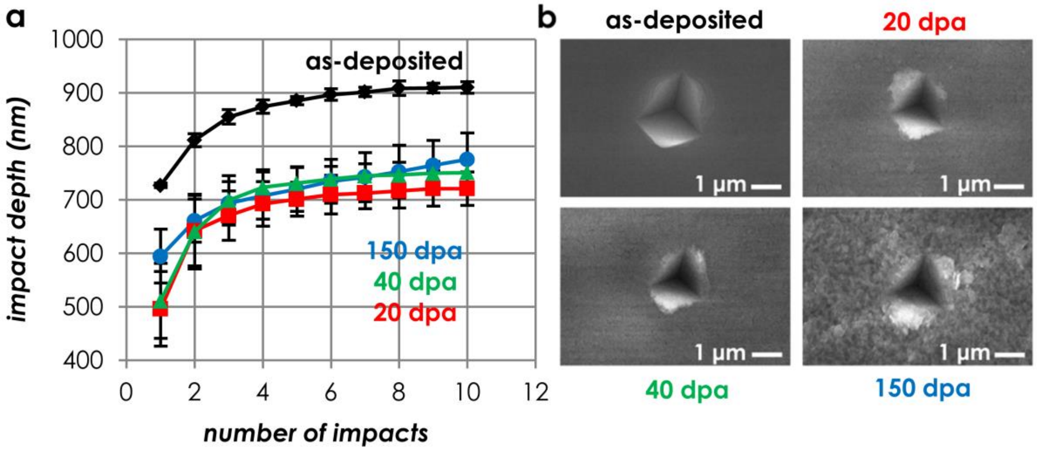

Figure S7. Displacement curves for nanoimpact testing of the Al_2O_3 nanoceramic at 1 mN before and after irradiation up to 20 dpa, 40 dpa and 150 dpa (a). The SEM micrographs on the right show the nanoimpact imprints (b).

The surface morphology of the nanoimpact imprints is examined by means of a field emission (FE) SEM microscope (Zeiss Supra 40). The electron voltage is set at 2 kV for imaging.
